# Sacral Stress Fracture following the Bone Union of Lumbar Spondylolysis

**DOI:** 10.1155/2016/9412315

**Published:** 2016-12-06

**Authors:** Tatsuro Sasaji, Hideki Imaizumi, Hiroyuki Takano, Hideo Saitoh, Taishi Murakami, Ryuichi Kanabuchi, Motohiko Sekiya

**Affiliations:** ^1^Department of Orthopedic Surgery, Osaki Citizen Hospital, 3-8-1 Furukawa Honami, Osaki-shi, Miyagi 989-6183, Japan; ^2^Sekiya Orthopedic Surgery, 5-1-19 Furukawa Ekimae Odori, Osaki-shi, Miyagi 989-6182, Japan

## Abstract

While 22 articles have reported on sacral stress fractures, it is a rare injury and its etiology is not well known. We present the case of a 16-year-old male who presented with low back pain in 2015. He was a high school soccer player with a previous history of a bilateral L5 lumbar spondylolysis in 2014. The patient refrained from soccer and wore a brace for six months. Two months after restarting soccer, he again complained of low back pain. After 1 year, a lumbar spine computed tomography revealed the bone union of the spondylolysis. At his first visit to our hospital, his general and neurological conditions were normal and laboratory data were within the normal range. Sacral coronal magnetic resonance imaging (MRI) of the left sacral ala revealed an oblique lineal signal void surrounding bone marrow edema. Based on his symptoms, sports history, and MRI, he was diagnosed with a sacral stress fracture. He again refrained from soccer; his low back pain soon improved, and, after 1 year, the abnormal signal change had disappeared on sacral MRI. Recurrent low back pain case caused by a sacral stress fracture occurring after the bone union of lumbar spondylolysis is uncommon.

## 1. Introduction

Lumbar spondylolysis is the most significant cause of low back pain in high school and college football players [1]. Lumbar spondylolysis is considered to be a form of stress fracture, the development of which is frequently associated with vigorous sports activities during the growth period [2]. In an early stage of lumbar spondylolysis, an orthosis leads to bone union [3].

Matheson et al. reported that, among 320 athletes with a bone scan-positive stress fracture, a pelvic fracture was reported in only five cases (1.6%) [4]. A sacral stress fracture was first reported by Czarnecki et al., and, to the best of our knowledge, 46 cases have been reported in 22 articles till date [5–26]. We present the case of a patient with a sacral stress fracture following the bone union of lumbar spondylolysis and report on the radiological findings and treatment course. We considered the two stress fractures (lumbar spondylolysis and sacral stress fracture) causing low back pain that happened in one patient to be a very rare condition. The patient and his family gave consent to submit these data for publication.

## 2. Case Report

**Figure 1:**
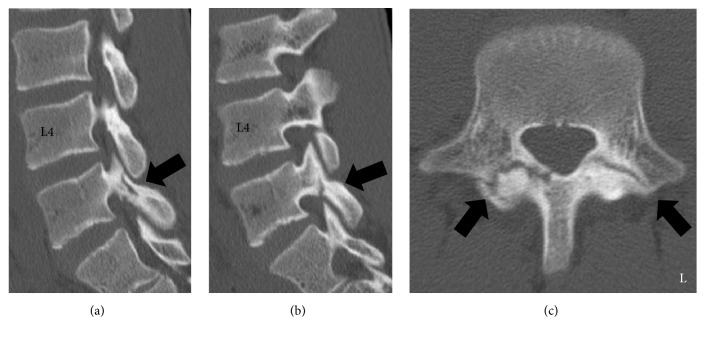
Reconstructed computed tomography (CT) scan of the lumbar spine from the first visit to a previous doctor's office. (a) Right parasagittal reconstructed CT image. (b) Left parasagittal reconstructed CT image. (c) Axial CT image at L5 level. Fissures (black arrows) in the bilateral pars interarticularis of the L5 can be seen (a, b, c). These radiological findings indicated a bilateral spondylolysis of the L5.

**Figure 2:**
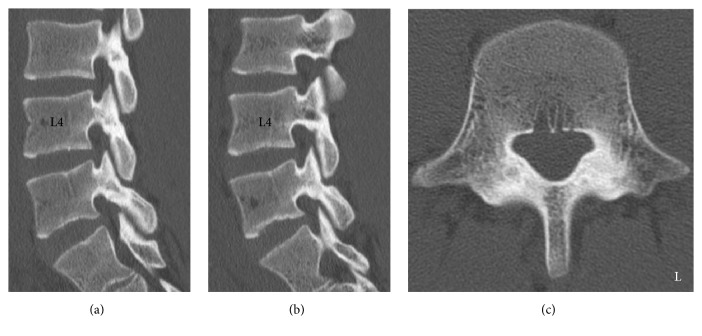
A 1-year follow-up reconstructed computed tomography (CT) scan of the lumbar spine after initial conservative treatment. (a) Right parasagittal reconstructed CT image. (b) Left parasagittal reconstructed CT image. (c) Axial CT image at L5 level. The radiological findings indicated that the bilateral spondylolysis of the L5 had united (a, b, c).

A 16-year-old male presented to our hospital with low back pain. He was a soccer player and had a history of lumbar spondylolysis that had been conservatively treated by a previous doctor. A period of rest and wearing a brace were advised in 2014. A lumbar computed tomography (CT) scan at his first visit to a previous doctor's clinic indicated that there were bilateral fracture lines in the L5 isthmus (Figures 1(a)–1(c)). His low back pain soon improved, and, after 10 months, he started playing soccer again in 2015. However, 2 months later, his low back pain recurred, and a 1-year follow-up lumbar CT indicated that his spondylolysis had united without displacement (Figures 2(a)–2(c)). He had no past history of malignant diseases or the use of steroid drugs.

### 2.1. Physiological Examination

The patient was 165 cm tall and weighed 50 kg. He complained of left sacral pain and had tenderness in the same region. A neurological examination at his first visit to our hospital indicated no neurological symptoms.

### 2.2. Laboratory Examination

Laboratory findings indicated a high alkaline phosphatase level and other tests were within normal limits (Table 1). There was no inflammatory reaction and infectious diseases were excluded.

### 2.3. Radiological Findings

**Figure 3:**
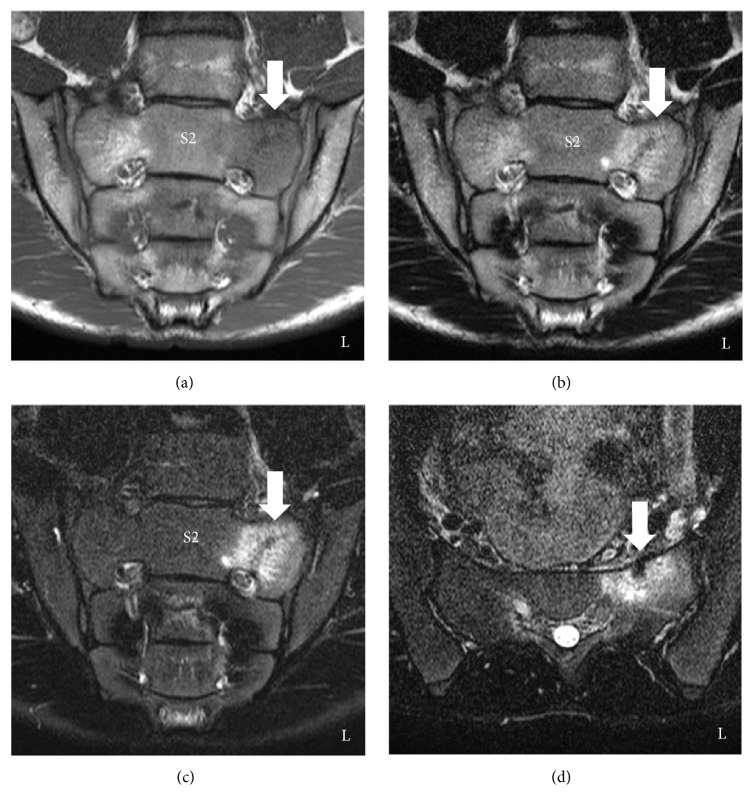
Magnetic resonance image of the sacrum at the first visit to our hospital. (a) Coronal plane on a T1-weighted image. (b) Coronal plane on a T2-weighted image. (c) Coronal plane on a short TI inversion recovery (STIR) image. (d) Axial plane at the S2 level on a STIR image. The left sacral ala on a T1-weighted image shows a low intensity (a), a T2-weighted image shows a high intensity (b), and on a STIR image shows a high intensity (c) compared with the right sacral ala. These radiological findings indicated bone marrow edema. Oblique lineal signal void (white arrows) can be seen in the bone marrow edema; these are fracture lines. An axial plane on a STIR image shows a low-intensity area (white arrow) surrounded by a high-intensity area in the left ventral surface of the sacral ala (d).

Sacral magnetic resonance imaging (MRI) obtained by the previous doctor showed a low-intensity T1-weighted image of the left second sacral ala and a high-intensity T2-weighted image and a short TI inversion recovery (STIR) image (bone marrow edema) compared with the right side. An oblique low lineal appearance could be seen in the bone marrow edema (Figures 3(a)–3(d)). On the basis of his sport history, laboratory data, past history, and radiological findings, the patient was diagnosed with a sacral stress fracture. Therefore, he was managed nonoperatively, and he discontinued soccer.

### 2.4. Treatment Course

**Figure 4:**
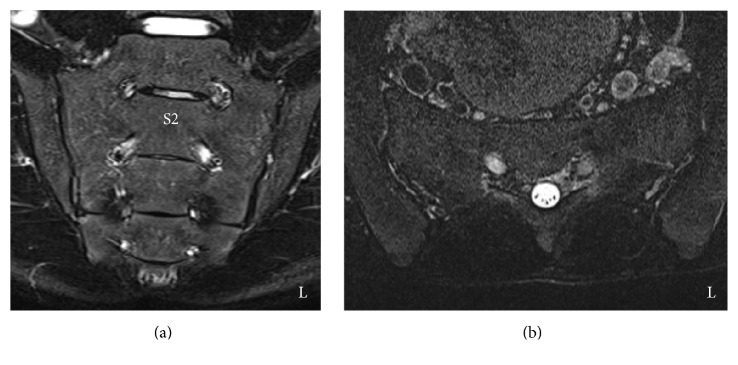
Magnetic resonance images of the sacrum one year after conservative treatment. (a) Coronal plane on a short TI inversion recovery (STIR) image. (b) Axial plane at the S2 level on a STIR image. Coronal plane (a) and axial plane at the S2 level (b) on a STIR image show no abnormal signal intensity. These radiological findings indicated bone union of the sacral stress fracture.

His lower back pain soon improved and within one year he had returned to activities of daily living without lower back pain. However, he retired from the soccer club for fear of a recurrent stress fracture. A 1-year follow-up sacral STIR image showed that the abnormal signal appearance in the left second sacral ala had disappeared (Figures 4(a) and 4(b)). Based on the fact that his symptoms had disappeared and on the radiological findings, we considered his sacral stress fracture as united.

## 3. Discussion

Kaneko et al. identified seven (2.3%) fractures in 311 sports-related low back pain cases [25]. We reviewed 46 detailed case reports of sacral stress fractures of which 15 cases were male and 31 cases were female (Table 2). The average age was 21.9 years (range 9–46 years). The right side was affected in 23 cases and the left side in 22 cases and one case was bilateral. Reported sports were long-distance running, cross-country running, basketball, and soccer. Our case can be included here as a proper sacral stress fracture.

In previous reports, the diagnosis of a sacral stress fracture was made based on a sports history and radiological findings. The reported main radiological methods were a bone scintigram and MRI. According to Grier et al., suggestive MRI findings of a fatigue fracture were a central lineal signal void on both T1- and T2-weighted sequences surrounded by diffuse low marrow signals on T1 images and increased signals on T2 images [8]. Previous studies that documented a sacral stress fracture reported MRI findings with a lineal low-intensity appearance in the high signal intensity area on T2-weighted sequences [8, 10, 11, 14, 17–20, 22–26]. The MRI findings of this case were compatible based on the radiological findings of previous reports.

While 22 articles have reported on sacral stress fractures, some authors reported a sacral stress fracture as an uncommon injury, although it is not rare based on the number of previous reports [5–26]. The common criteria regarding a sacral stress fracture were young healthy athletes, MRI and/or bone scintigraphy as a diagnostic tool, and pain relief upon cessation of sports [5–26]. Based on the previous reports, we considered that complicated diagnosis and easy improvement by rest left sacral stress fracture not diagnosed and made it a not well-known disease for orthopedic doctors.

In our review of 37 cases of sacral stress fracture including descriptions of a past stress fracture (Table 3), five cases had a previous history of a stress fracture [10, 12, 14, 17, 18]. Three cases involved the tibia, one involved the metatarsal and tibia, and one involved the sacrum [10, 12, 14, 17, 18]. To our knowledge, this case is the first report of a sacral stress fracture after union of a lumbar spondylolysis and we consider it to be a rare pathological condition.

## Figures and Tables

**Table 1:** Laboratory data.

White blood cell (/*μ*L, 33–81)	4540
Alkaline phosphatase (U/L, 115–359)	494
Lactate dehydrogenase (U/L, 119–229)	189
Sodium (mEq/L, 138–146)	144
Chlorine (mEq/L, 99–109)	104
Potassium (mEq/L, 3.6–4.9)	4
Calcium (mg/dL, 8.7–10.3)	9.4
Phosphorus (mg/dL, 2.5–4.7)	4.2
Creatine kinase (U/L, 62–287)	120
C-reactive protein (mg/dL, 0–0.3)	0.03

**Table 2:** Review of reports.

Reference	Side	Sex	Sports	Age (year)
[5]	Left	Male	Long-distance runner	26
[6]	Right	Female	Runner	28
[7]	Right	Male	Long-distance runner	40
[8]	Left	Female	Runner	14
[8]	Left	Male	None	9
[9]	Left	Female	Cross-country and distance runner	21
[9]	Right	Female	Cross-country and distance runner	21
[9]	Left	Female	Multiple sports	17
[10]	Right	Female	Cross-country runner	20
[10]	Right	Female	Long-distance runner	21
[10]	Right	Female	Cross-country runner	20
[11]	Right	Male	Basketball	20
[12]	Left	Male	Long-distance runner	19
[13]	Right	Male	Runner	28
[14]	Left	Female	Soccer	21
[14]	Left	Female	Basketball	20
[14]	Left	Female	Runner	45
[14]	Left	Female	Cross-country runner	22
[14]	Left	Female	Jogging	41
[14]	Right	Female	Jogging	19
[14]	Right	Female	Cross-country runner	20
[14]	Left	Female	Cross-country runner	21
[15]	Left	Female	Volleyball	16
[16]	Left	Male	Long-distance runner	21
[17, 18]	Left	Male	Long-distance runner	20
[17, 18]	Left	Female	Long-distance runner	21
[17, 18]	Left	Female	Long-distance runner	21
[17, 18]	Right	Female	Long-distance runner	20
[17, 18]	Right	Female	Long-distance runner	21
[19]	Right	Female	Tennis	46
[20]	Right	Female	Runner	21
[20]	Right	Female	Cross-country runner	18
[21]	Left	Male	Long-distance runner	26
[21]	Left	Male	Runner	23
[22]	Left	Female	Basketball	16
[22]	Left	Male	Long-distance runner	17
[23]	Right	Female	Marathon	34
[24]	Right	Male	Professional hockey	27
[25]	Right	Male	Soccer	15
[25]	Right	Female	Soccer	18
[25]	Right	Female	Softball and athletic sports	10
[25]	Right	Female	Softball	15
[25]	Bilateral	Male	Basketball	14
[25]	Right	Female	Basketball	16
[25]	Left	Male	Baseball	15
[26]	Right	Female	Mounted police officer	26

**Table 3:** Review of reports.

Reference	A history of stress fracture
[5]	None
[7]	None
[8]	None
[8]	None
[9]	None
[9]	None
[9]	None
[10]	None
[10]	Right sacrum
[10]	None
[11]	None
[12]	Right tibia
[13]	None
[14]	Metatarsal, bilateral tibia
[14]	None
[14]	None
[14]	None
[14]	None
[14]	None
[14]	None
[14]	None
[15]	None
[16]	None
[17, 18]	None
[17, 18]	Bilateral tibia
[17, 18]	Right tibia
[17, 18]	None
[17, 18]	None
[20]	None
[20]	None
[21]	None
[21]	None
[22]	None
[22]	None
[23]	None
[24]	None
[26]	None
